# Inhibitory Effect of *Helicteres gardneriana* Ethanol Extract on Acute Inflammation

**DOI:** 10.1155/2012/141947

**Published:** 2011-10-19

**Authors:** Juliana Oliveira de Melo, Laura Lícia Milani de Arruda, Silmara Baroni, Maria da Conceição Torrado Truiti, Silvana Martins Caparroz-Assef, Roberto Kenji Nakamura Cuman, Ciomar Aparecida Bersani-Amado

**Affiliations:** ^1^Laboratory of Inflammation, Department of Pharmacology and Therapeutics, State University of Maringá, Maringá, PR, Brazil; ^2^Laboratory of Pharmaceutical Technology, Department of Pharmacy, State University of Maringá, Maringá, PR, Brazil

## Abstract

The anti-inflammatory effect of an ethanol extract of *Helicteres gardneriana* (Nees) Castiglioni was assayed in experimental models of pleurisy and microcirculation *in situ*. Treatment of animals with 500 mg/kg body weight reduced the exudate volume (35% reduction) induced by intrapleural injection of carrageenan and the migration of polymorphonuclear cells into the inflamed pleural cavity of rats (40%). Additionally, rolling and adhesion of leukocytes and the number of leukocytes that migrated toward the perivascular space in response to the carrageenan injection were decreased by the extract (500 mg/kg). These data demonstrate the anti-inflammatory effect of the ethanol extract of *Helicteres gardneriana* and imply that inhibition of leukocyte-endothelial interactions is important in the extract's mechanism of action.

## 1. Introduction

The genus *Helicteres* belongs to the family Sterculiaceae and includes several species such as *H. angustifolia, H. sacarolha*, and *H. isora* that are used in folk medicine for their analgesic and anti-inflammatory properties [1–3]. Studies performed with extracts obtained from *H. isora* have demonstrated its ability to inhibit abdominal writhing induced by administration of acetic acid in a mouse model of pain [4].

Plants of the genus *Helicteres *(*H. isora* and *H. angustifolia*) contain triterpenoids [5, 6], cucurbitacins [7, 8], flavonoids [9, 10], neolignans [11], rosmarinic acids [12], and essential oils [13] which may contribute to their pharmacological effects. *In vivo *and* in vitro* experimental assays have shown that some of these constituents have anti-inflammatory effects [14–19]. 

Recent experimental assays have shown that applying *Helicteres gardneriana* crude extract significantly reduced ear edema and inhibited the activity of the enzyme myeloperoxidase. Furthermore, photoacoustic data showed that the strong anti-inflammatory effects of the extract were associated with the deep penetration observed for the extract [20]. In spite of these findings, no studies have evaluated the systemic anti-inflammatory effects of *Helicteres gardneriana* extract.

The acute inflammation reaction is characterized by edema formation and recruitment of leukocytes into the injured tissue. At the beginning of the inflammatory process, chemical substances secreted at the site of injury mediate the endothelial cell contraction, causing a subsequent increase in vascular permeability and consequent edema formation. Simultaneously, a coordinated sequence of events occurs and initiates the migration of leukocytes from the vascular system to the site of the lesion. Tethering and rolling of leukocytes on the vessel wall is the initial and fundamental event, followed by firm adhesion to the endothelium [21, 22]. The inflammatory reaction is necessary for tissue recovery and provides the correct cytokine signals and cell machinery to clear up the site for tissue regeneration [23]. However, uncontrolled inflammation has unfavorable effects on the course of tissue healing because the inflammatory cells are also able to induce tissue damage.

The present study evaluated the effects of an ethanol extract of *Helicteres gardneriana* (EEHg) on the inflammatory response using models of carrageenan-induced pleurisy and microcirculation *in situ*. Here, we describe the anti-inflammatory effects of this extract on the edema, mobilisation of leukocytes, and leukocyte-endothelium interactions.

## 2. Materials and Methods

### 2.1. Animals

Male Wistar rats (220–260 g) were used. The animals were maintained in a controlled temperature of 22°C and a 12 h light/dark cycle with water and food available *ad libitum*. The experimental protocol was approved by the Ethics Committee for Animals of the State University of Maringá (no. 024/2006).

### 2.2. Plant Material

The aerial parts of the *Helicteres gardneriana* plant were collected in July 2004 in the floodplain of the Upper Paraná River, Municipality of Taquaraçú, Mato Grosso de Sul, Brazil. The material was appropriately preserved and deposited at the Nupélia Herbarium of the State University of Maringá, Paraná, Brazil (HNUP no. 2844). The material was dried in an air-circulating oven at 40°C and then ground in a cutting mill. The extract was obtained by extraction with absolute ethanol at room temperature. The solvent was then removed in a rotating evaporator to produce the ethanol extract [24]. Immediately prior to use, the EEHg was diluted in a 16% dimethylsulfoxide solution (DMSO : water, 1 : 6).

### 2.3. Rat Pleurisy

Pleurisy was induced by injection of 0.25 mL of a carrageenan suspension (200 *μ*g) in the intrapleural cavity, according to the technique described by Vinegar et al. [25]. The carrageenan was diluted in saline buffered with phosphate (PBS, pH = 7.4). Four hours after induction of pleurisy, the animals were killed, and the inflammatory exudate was collected. The exudate volume was measured, and an aliquot of 50 *μ*L was diluted in Turk solution (1 : 20) and used to determine the total number of leukocytes in a Neubauer chamber. For differential counting of leukocytes, the remaining fluid was centrifuged at 2500 rpm for 10 min, and the cells were resuspended. The slides were prepared, dried, fixed, and stained with May-Grunwald-Giemsa. The number of mononuclear and polymorphonuclear leukocytes in the exudate was determined with the aid of a light microscope. The EEHg (250 and 500 mg/kg), dexamethasone (0.5 mg/kg—standard antiinflammatory), vehicle (16% DMSO—negative control), and saline (0.9%—negative control) were administered orally by gavage, in different groups of rats, which had fasted for 15 h, 30 min prior to the induction of pleurisy.

### 2.4. Determination of Total Nitric Oxide Concentration in Pleural Exudates

The concentration of total nitric oxide (NO_3_^−^ + NO_2_^−^) was determined in the pleural exudates of control rats and rats treated with EEHg. The samples were deproteinized by centrifugation (5000 ×g for 120 min at 4°C) in Eppendorf tubes with 10 kDa filters. Total NO was determined by first incubating the samples and calculating the standard curve (aqueous solutions of KNO_3_ at concentrations ranging from 0.2 to 200 *μ*M) with 20 *μ*L nicotinamide adenine dinucleotide phosphate (100 *μ*M), flavin adenine dinucleotide (5 *μ*M), and NO_3_^−^ reductase (200 *μ*m/mL) for 1 h at 37°C. Next, 20 *μ*L lactate dehydrogenase (13.5 U/mL) and pyruvate (9 mM) were added, and the samples were further incubated for 30 min at 37°C. After this period, 50 *μ*L of Griess reagent (a solution containing 0.1% sulfanilamide +0.01% naphthylethylenediamine in 5% phosphoric acid) was added, and the samples were allowed to rest for 10 min at ambient temperature. The concentrations of total NO were determined in the exudate by measuring absorbance at 540 nm [26].

### 2.5. Determination of Rolling, Adhesion, and Migration of Leukocytes in the Microcirculation of Spermatic Fascia in situ

Rolling behavior and adhesion of leukocytes to the endothelium were evaluated in the internal spermatic fascia of rats 2 h after injection of the carrageenan suspension (100 *μ*g) in the wall of the scrotal chamber [27, 28]. Animals anesthetized with chloral hydrate (500 mg/kg, s.c.) were maintained on a special board thermostatically controlled at 37°C with a transparent platform for transillumination of the tissue on which the spermatic fascia was exposed and fixed for microscopic analysis* in situ*. The preparation was kept moist and warm with Ringer-Locke's solution (pH 7.2–7.4) containing 1% gelatin. The vessels selected for the study were postcapillary venules with a diameter of 15–25 *μ*m. The numbers of rolling and adherent leukocytes were determined at 10 min intervals. The leukocytes were considered to adhere to the venular endothelium if they remained stationary for more than 30 s. In another series of experiments, the number of leukocytes that migrated to an area of 2500 *μ*m^2^ of connective tissue adjacent to the postcapillary venules 4 h after carrageenan injection was determined. This area was defined on the video screen, 80 × 32 *μ*m in tissue corresponding to 9.2 × 3.7 cm on the screen. Five different fields were evaluated on a single animal to avoid variability based upon sampling. Data were then averaged for each animal. EEHg (500 mg/kg), dexamethasone (0.5 mg/kg), vehicle (16% DMSO), and saline (0.9%) were administered orally by gavage 30 min before the carrageenan injection to different groups of rats that were fasted for 15 h.

### 2.6. Statistical Analysis

Results are expressed as mean ± standard error of the mean (S.E.M.). Data were subjected to analysis of variance (one-way ANOVA) followed by Tukey's *post hoc* test. Values of *P* < 0.05 were considered statistically significant.

## 3. Results

### 3.1. Effect of EEHg on Pleurisy

Intrapleural injection of carrageenan in groups of animals pretreated orally with saline or DMSO induced an acute inflammatory response, characterized by an increase in pleural exudate volume and number of leukocytes migrated to the cavity, compared to basal parameters (obtained from normal animals that received an injection of PBS in the cavity) (Vol. exudate: basal = 0.1 mL; Cg + Saline = 0.91 ± 0.07 mL; Cg + DMSO = 0.9 ± 0.07 mL; number of leukocytes/mm^3^: basal = 6700 ± 450; Cg + Saline = 59800 ± 4810; Cg + DMSO = 60270 ± 3910). Treatment of animals with EEHg (500 mg/kg, p.o., administered 30 min prior to carrageenan injection) significantly reduced the volume of the inflammatory pleural exudate (31%  *P* < 0.001) and the number of migrated polymorphonuclear leukocytes (30%  *P* < 0.001). Treatment of the animals with dexamethasone caused a pronounced reduction in the volume of the exudate (73%  *P* < 0.001) and in the number of migrated leukocytes (53%  *P* < 0.001). The results are presented in Table 1. 

### 3.2. Effect of EEHg on the Total Concentration of Nitric Oxide Present in Pleural Exudates

As expected, NO levels increased in the inflammatory pleural exudate of control rats pretreated with 0.9% saline or 16% dimethylsulfoxide, p.o., 30 min before intrapleural carrageenan injection (Basal, 10.1 ± 1.2 *μ*M; Cg + Sal, 32.5 ± 2.3 *μ*M; Cg + DMSO, 31.6 ± 1.8 *μ*M). Treatment of animals with the EEHg did not significantly change NO concentrations in inflammatory pleural exudates (Cg + Hg_500_, 28.1 ± 1.1 *μ*M).

### 3.3. Effect of EEHg on Rolling, Adhesion, and Migration of Leukocytes in the Microcirculation of Spermatic Fascia In Situ

Intradermal injection of carrageenan into the wall of the scrotal chamber of animals pretreated orally with 0.9% saline (Cg + Sal) or 16% dimethylsulfoxide (Cg + DMSO) induced an acute inflammatory response characterized by an increase in rolling leukocytes, adherence of leukocytes to vascular endothelial, and migration of leukocytes to the perivascular space 2 and 4 h after induction of a local inflammatory reaction compared with the baseline (obtained from normal animals that received saline injection in the scrotal chamber). Treatment of animals with EEHg (500 mg/kg) reduced the number of rolling leukocytes (84.0%) and the number of adherent leukocytes (90.7%) 2 h after stimulation with carrageenan. The same treatment reduced the number of leukocytes that migrated to the tissue adjacent to postcapillary venules (90.5%) 4 h after stimulation with carrageenan. Dexamethasone caused total inhibition of the number of rolling and adherent leukocytes and almost complete inhibition of the number of migrated leukocytes (97%; Table 2).

## 4. Discussion

The search for new drugs that effectively interfere with the inflammatory process continues to be important. In the present study, the anti-inflammatory activity of orally administered EEHg was evaluated in models of carrageenan-induced pleurisy and microcirculation *in situ* by injecting carrageenan into the scrotal chamber of rats.

The model of pleurisy has been extensively used to investigate the mechanisms involved in acute inflammation and to evaluate the effectiveness of drugs with anti-inflammatory properties [25, 29]. As expected, in our experiments, intrapleural carrageenan injection caused an accumulation of pleural exudate, accompanied by intensive migration of inflammatory cells into the pleural cavity. Oral administration of the EEHg significantly reduced the volume of pleural exudate that accumulated in response to carrageenan injection. Furthermore, treatment with the extract also inhibited the migration of polymorphonuclear cells but was not effective at reducing the number of mononuclear cells in the pleural cavity. Similarly, in previous studies, we found that the EEHg was also able to strongly reduce the migration of neutrophils in a mouse model of ear edema induced by croton oil, demonstrated by a reduction in myeloperoxidase activity [20].

The pronounced effect of the EEHg in experimental models of inflammation encouraged us to investigate its effect on the process of leukocyte migration to the inflammatory site. To our knowledge, few studies have demonstrated the inhibitory actions of plant extracts on leukocyte-endothelial interactions in microcirculation [30]. This process is complex and involves a sequence of molecular-mechanical events on leukocyte and endothelial cells, including rolling, arrest, adhesion, and transendothelial diapedesis. These events require reciprocal interactions between molecules in neutrophils and endothelial cells, in addition to the production of inflammatory and chemotactic mediators that modulate the recruitment of neutrophils [31].

In the present study, the data obtained show that the EEHg markedly inhibits the rolling behavior of leukocytes, their subsequent firm adherence to the vessel wall, and the cell migration after application of an inflammatory stimulus. Therefore, the inhibitory effect of EEHg on the process of exudation (edema) and cell migration suggests that it might exert effects on the chemical mediators involved in the vascular response and the chemotaxis process. Additionally, neutrophils adhering to the vessel wall secrete a number of enzymes and chemical mediators that contribute to edema formation [32, 33], which is consistent with subsequent studies that have demonstrated impairment of the acute increase in vascular leakage in animals depleted of neutrophils [34, 35].

Therefore, we hypothesized that the impairment of neutrophil interactions with the vessel wall contributes to reduced edema in animals treated with EEHg, indicating that the interaction of leukocytes with the vessel wall is an important mechanism underlying its anti-inflammatory effects. Importantly, the anti-inflammatory effect of EEHg extract was observed in different tissues, possibly involving an inhibitory effect on different chemical mediators, and thereby suggesting systemic activity. However, we could not precisely determine which mediator or proinflammatory agent is inhibited by the extract. In the pleurisy model, the possibility of an effect on NO generation may be discarded because EEHg did not alter total NO concentration in the pleural exudate. Experiments are underway to assess the mechanism by which the active components of the *Helicteres gardneriana* extract exert their anti-inflammatory effects.

## Figures and Tables

**Table 1:** Exudate volume and total and differential leukocytes counts in inflammatory pleural Exudate of rats, 4 h after injection of carrageenan (Cg, 200 *μ*g/cavity).

Groups of animals	Exudate volume (mL)	Leukocytes/mm^3^		
		Total	mononuclear	Polymorphonuclear
Basal	0.10 ± 0.01	6700 ± 450	1800 ± 160	4900 ± 390
Cg + Sal	0.91 ± 0.07^c^	59800 ± 4810^c^	11770 ± 107^c^	48030 ± 4176^c^
Cg + DMSO	0.90 ± 0.07^c^	60270 ± 3910^c^	9850 ± 1333^c^	50420 ± 2999^c^
Cg + Dex_0.5_	0.24 ± 0.01^e^	28000 ± 1447^a,e^	2309 ± 324^b,e^	25691 ± 1355^b,e^
Cg + EEHg_250_	0.85 ± 0.10^c^	58090 ± 3986^c^	9040 ± 1361^c^	49050 ± 3059^c^
Cg + EEHg_500_	0.63 ± 0.04^c,d^	41860 ± 3434^c,d^	13080 ± 1409^c^	28780 ± 2573^b,d^

Each value represents the mean ± S.E.M. of 6–8 animals. Dexamethasone (Dex) administered orally, 0.5 mg/Kg was used as a reference anti-inflammatory (positive control). Basal = animals that received injection of PBS in the cavity (PBS + Sal), Cg = carrageenan, Sal = 0.9% saline, DMSO = 16% dimethylsulfoxide, EEHg = ethanol extract of *Helicteres gardneriana*. ^a^ *P* < 0.05, ^b^ *P* < 0.01, and ^c^ *P* < 0.001, compared to basal group, ^d^ *P* < 0.05, ^e^ *P* < 0.001 compared to groups Cg + Sal and Cg + DMSO (one-way ANOVA, Tukey's test).

**Table 2:** Number of rolling and adherent leukocytes during 10 min periods after 2 h and migrated leukocytes after 4 h of inflammatory stimulus.

Groups of animals	*n*	Leukocytes		
		Rolling	Adherent	Migrated
Basal	5-6	126.8 ± 10.2	7.8 ± 1.4	7.7 ± 0.3
Cg + Sal	5–9	205.1 ± 14.0^a^	23.0 ± 1.9^a^	17.4 ± 0.8^a^
Cg + DMSO	5–7	201.3 ± 14.8^a^	22.0 ± 0.9^a^	17.0 ± 0.4^a^
Cg + Dex	5-6	110.8 ± 11.2^b^	6.3 ± 0.7^b^	7.9 ± 0.2^b^
Cg + EEHg_500_	6-7	138.7 ± 11.2^b^	9.2 ± 1.3^b^	8.6 ± 0.5^b^

Each value represents the mean ± S.E.M. of 6–8 animals. Dexamethasone (Dex) administered orally, 0.5 mg/Kg was used as a reference anti-inflammatory (positive control). Basal = animals that received injection of saline in the scrotal pouch (Sal + Sal), Cg = carrageenan, Sal = 0.9% saline, DMSO = 16% dimethylsulfoxide, EEHg = ethanol extract of *Helicteres gardneriana*. ^a^ *P* < 0.05, compared to basal group. ^b^ *P* < 0.001, compared to the groups Cg + Sal and Cg + DMSO (one-way ANOVA, Tukey's test).
